# Knowledge and awareness of rare diseases among healthcare professionals: a scoping review

**DOI:** 10.1186/s13023-026-04520-1

**Published:** 2026-08-13

**Authors:** Stacey McGeown, Paul Slater, Felicity Hasson

**Affiliations:** 1 Institute of Nursing and Health Research, School of Nursing & Paramedic Science, Ulster University, York Street, Belfast, BT15 1AP Northern Ireland; 2 Queen’s University Belfast, Belfast, Northern Ireland

**Keywords:** Knowledge, Awareness, Healthcare professional, Medical education, Continuing education, Rare disorder, Rare developmental defect, Rare genetic disorder, Scoping review

## Abstract

**Background:**

Rare diseases affect millions globally but remain poorly understood due to low prevalence and fragmented healthcare responses. Patients face long diagnostic delays, known as the “diagnostic odyssey”, due to limited knowledge and awareness among healthcare professionals. This scoping review examines healthcare professionals’ knowledge and awareness of rare diseases. Findings highlight critical educational and systemic gaps across disciplines. Addressing these deficits is essential to improve early recognition, care coordination, and health outcomes for rare disease populations.

**Methods:**

This scoping review followed Arksey and O’Malley’s five-stage framework, enhanced by the Joanna Briggs Institute (JBI) methodology and PRISMA-ScR guidelines. The review mapped existing literature guided by the PCC framework. A comprehensive search strategy was developed and executed across four databases, CINAHL, Medline, PubMed, and Scopus, along with grey literature sources. After removing duplicates, 4539 records were screened, and 25 studies met the inclusion criteria. Data was charted and synthesised thematically using an inductive approach to identify key findings and gaps in professional education and awareness.

**Results:**

Twenty-five studies from 2015 to 2025 were included, spanning diverse countries and healthcare professions. Most used cross-sectional surveys to assess knowledge, awareness, and confidence regarding rare diseases. Overall, healthcare professionals demonstrated limited knowledge, poor familiarity with rare disease definitions, and low use of specialist resources. Variability was observed across professional groups, with general practitioners and nurses demonstrating lower knowledge than specialists. Education and training were widely reported as insufficient, with gaps evident across both undergraduate and clinical settings. Despite low objective knowledge, many participants overestimated their competence. These knowledge gaps were associated with challenges in diagnosis and management, including delayed recognition of rare diseases. A strong demand for improved rare disease education and accessible guidelines was consistently expressed.

**Conclusion:**

Despite growing recognition of rare diseases as a public health priority, healthcare systems worldwide lack structured education, standardised guidelines, and accessible resources. The review calls for further research, urgent educational reform, improved digital resources, and targeted policy changes to integrate rare disease education into healthcare curricula and enhance clinical support. Addressing these gaps will improve care, facilitate earlier diagnoses, and ensure more equitable outcomes for rare disease patients.

**Supplementary Information:**

The online version contains supplementary material available at https://doi.org/10.1186/s13023-026-04520-1.

## Background

Rare diseases (RD) though individually infrequent, are common in the aggregate, and collectively encompass over 7,000 conditions, representing a significant proportion of the global population [1]. Globally, this is estimated at 260–440 million people. In the European Union, approximately 36 million people live with rare diseases, with about 3.5 million, or 1 in 17, affected in the UK alone [2, 3]. This prevalence is strikingly comparable to diabetes, a well-recognised public health priority, affecting 1 in 16 people [4].

Most are genetic in origin and associated with reduced life expectancy and quality of life [5]. These include rare cancers, bleeding disorders, rare infections, and more familiar conditions such as cystic fibrosis [6]. The accurate diagnosis, treatment and management of these conditions is a major challenge for healthcare professionals (HCP), who do not have enough knowledge to identify them [7]. Given the sheer number and diversity of rare conditions, it is unfeasible for HCP to receive comprehensive training in all of them. Despite the rarity of each condition, it is only the condition that is rare. The overall burden for patients, carers and families is common across the landscape. Ensuring broad awareness, integrating rare disease modules into curricula, and promoting interprofessional education are vital to improving patient outcomes, quality of life and reducing diagnostic delays [8].

## Introduction

There is no universally accepted definition of rare diseases. A review by Richter et al. [9] identified over 300 definitions worldwide. Most definitions share a common criterion: low prevalence. These conditions place a heavy burden on patients and families, as well as on HCP who often face difficulty diagnosing and managing them [10, 11].

Approximately 75% of rare diseases manifest in childhood, and over 30% of affected children die before the age of five [12]. The distribution and frequency of individual diseases can vary significantly for example, Creutzfeldt–Jakob disease affects approximately 1 in 1 million individuals annually, whereas Huntington’s disease occurs in 5 to 10 per 100,000 among people of European descent [13].

Although symptom presentations vary, people living with RD share similar barriers. The most significant of these is the challenge of diagnosis. Many patients remain undiagnosed or misdiagnosed, leading to what is commonly called the “diagnostic odyssey,” which averages five years [14]. During this time, patients often undergo numerous consultations and unnecessary or even harmful treatments [15]. Diagnostic delays are often due to a lack of awareness, insufficient training, and poorly coordinated healthcare systems [16].

Primary care providers frequently miss early signs due to limited knowledge or lack of access to specialist referrals [17]. This leads to frustration for patients, families, and providers alike [18]. In addition to medical challenges, patients face social exclusion, financial hardship, and emotional distress [19]. Most RD are chronic or progressive, with limited or no effective treatments [20]. General practitioners (GPs), emergency clinicians, and community-based providers rarely receive sufficient training in RD, further limiting early recognition [1]. Nurses, pharmacists, and allied health professionals face similar deficits [21].

Family members and caregivers experience heightened psychological distress, economic burdens, and reduced quality of life [22]. Out-of-pocket costs, travel for specialist care, and the need to reduce working hours or leave employment are common [23, 24]. The lack of a multidisciplinary approach and limited availability of specialists and treatments further compounds these difficulties [11]. Fewer than 5% of RD currently have approved therapies, largely because pharmaceutical companies lack financial incentives to develop treatments for small patient populations [25, 26].

Undergraduate and postgraduate education on RD remains limited [27], and clinicians are often left without the skills or confidence to manage rare presentations [28]. These gaps contribute to diagnostic delays, fragmented care, and poor communication [28–30]. Although some schools are starting to incorporate RD into training, implementation is inconsistent [31]. There have been recent education initiatives, such as the UK charity Medics4Rares “Rare Disease 101” which offers an online course for clinicians and medical students. More recently they have developed E-learning for nurses in partnership with GNNRD (Global Nursing Network for Rare Disease), this has received positive initial feedback, though further evaluation is needed [32].

Rare diseases present a growing public health challenge and pose complex medical, emotional, and social issues. Improving awareness and knowledge across all healthcare sectors is critical. This comprehensive scoping review of healthcare professional knowledge and awareness will synthesise the literature to better understand the gaps and inform future education, clinical practice and policy development.

## Methods

### Study design and research questions

Given the broad scope and exploratory aim, a scoping review was deemed the most appropriate methodology [33–35]. This review was conducted in accordance with the framework proposed by Arksey and O’Malley, which includes five stages: identifying the research question, identifying relevant studies, study selection, charting the data, and collating and reporting the results [36]. The review was further guided by the Joanna Briggs Institute (JBI) methodology for scoping reviews and reported in accordance with the PRISMA-ScR checklist [37]. This scoping review was not registered.

The review question was developed using the Population–Concept–Context (PCC) framework: Population **–** healthcare professionals; Concept – knowledge and awareness; Context – rare diseases. The review was guided by the question “What is the level of healthcare professionals’ knowledge and awareness of rare diseases?”

### Search strategy

The literature search was conducted by the first author (SG) following development of the search strategy in consultation with a specialist subject librarian. Independent review by two reviewers (SM and FH) was applied during the study selection and data extraction stages.

A comprehensive search strategy was developed and conducted in accordance with Joanna Briggs Institute (JBI) guidance [37] (Additional file 1). A three-step search process was employed. First, an initial limited search of PubMed was undertaken to identify relevant keywords and index terms. Second, a comprehensive search using these terms was conducted across multiple databases. Third, the reference lists of included studies were screened to identify additional relevant articles.

The following databases were searched: PubMed/MEDLINE (via PubMed), CINAHL (EBSCOhost; 1982–March 2026), and Scopus (Elsevier; 1960–March 2026). Searches were conducted from database inception to March 2026, with results limited to studies published in English between March 2015 and March 2026.

Search terms were developed using the PCC framework, and based on three core concepts: healthcare professionals, knowledge and awareness, and rare diseases (Additional file 2). Keywords and synonyms were combined using Boolean operators, with terms within each concept linked using “OR” and concepts combined using “AND”. Truncation and phrase searching were applied to capture variations in terminology and improve search sensitivity [38, 39]. The search strategy was iteratively refined to ensure relevance and comprehensiveness.

Grey literature was also searched using Google Scholar and relevant organisational websites. These websites included the Department of Health and Social Care, Orphanet, and EURORDIS [40, 41, 5].

Forward citation searching was also conducted using Google Scholar for all included studies. Titles and abstracts of citing articles were screened against a pre-defined inclusion/exclusion criteria (Additional file 3).

### Study selection

All search results were imported into Covidence for screening and duplicate removal. A total of 7,495 records were identified, of which 2,596 duplicates were removed. The remaining 4,539 titles and abstracts were screened against the inclusion and exclusion criteria.

Study selection was conducted in two stages by two independent reviewers (SG and FH) to minimise selection bias. First, titles and abstracts were screened for relevance. Second, full-text articles were assessed for eligibility. Disagreements at any stage were resolved through discussion, with a third reviewer consulted where necessary.

Eligibility criteria included studies involving healthcare professionals and reporting outcomes related to knowledge or awareness of rare diseases. Studies focusing exclusively on students, specific rare diseases, or treatment/management without addressing knowledge or awareness were excluded.

Thirty-eight full-text articles were assessed for eligibility and were screened against a predefined inclusion and exclusion criteria − 25 met the final criteria. Study selection followed PRISMA-ScR guidelines, with reasons for exclusion recorded at each stage to ensure transparency and reproducibility. The study selection process is illustrated in the PRISMA-ScR flow diagram (Additional File 4).

### Data extraction

To enhance transparency, a standardised data extraction table (Additional file 5) was developed and used based on the JBI framework capturing, core study details, including design, participants, methodology, outcomes, and main results [42].

Data extraction was conducted independently by two reviewers (FH and SG). Any discrepancies were resolved through discussion to ensure accuracy and consistency. This approach minimised the risk of bias and human error during data synthesis [43].

### Data analysis

Scoping reviews typically aim to map the breadth of evidence rather than assess its quality or effect size [44, 45]. Considering this, a narrative synthesis of the data was used. This helped to provide a flexible and transparent means of summarising the findings, especially when the data is too heterogeneous for quantitative aggregation [46, 47]. It enabled the identification of key themes, patterns, and knowledge gaps in the literature [35]. A narrative approach allowed for the integration of both qualitative and quantitative data, making it a flexible approach for summarising and interpreting research findings. Following this, themes were identified and explored using subheadings.

## Results

A total of 25 studies conducted between 2015 and 2025 were identified, of which 24 were primary cross-sectional studies and one was a systematic review. These studies covered a broad geographical and professional spectrum. Studies were conducted across 17 countries spanning Europe (Spain, Belgium, Germany, Poland, Italy, Malta), Asia (China, India, Kazakhstan, Turkey), the Middle East (Bahrain, Israel), Africa (Cameroon), South America (Peru, Mexico), and global multi-country settings [16, 28, 48–72]. 

The included studies predominantly used quantitative cross‑sectional survey designs utilising non-validated instruments, with questionnaire lengths varying from 14 to 102 items and formats incorporating both closed and open‑ended responses. Two studies implemented dual surveys targeting healthcare professionals and the public [57, 60]. One study adopted a multi‑method approach combining surveys with expert interviews [70], while one study was an evidence synthesis conducted as a systematic review across five databases (2014–2024) [65].

Across the included studies, data were drawn from diverse populations comprising physicians, nurses, medical and nursing students, dentists, neurologists, emergency physicians, allied health professionals, rare disease experts, members of the public, and large clinician panels,

Sample sizes varied considerably, ranging from small qualitative cohorts (*n* = 9) to national surveys exceeding 69,000 clinicians. However, many studies were conducted within single institutions or specific regions, limiting the generalisability of findings [72].

Across studies, outcomes assessed knowledge, awareness, attitudes, and educational needs related to RD. Measures included self‑assessed and objective knowledge levels, awareness of RD prevalence and characteristics, preparedness for diagnosis and care, and accuracy in recognising RD features. Studies also examined information‑seeking behaviours, utilisation of RD information platforms, perceptions of RD education and training needs, awareness of RD‑related policies, and views on RD as a public health priority. Additional outcomes captured diagnostic challenges, frequency of RD encounters, identification of commonly seen RD, and factors associated with knowledge. Public‑facing studies assessed awareness, experience, and attitudes toward RD and orphan medicines, while some studies explored research prioritisation and resource allocation for RD.

Across the included literature, knowledge and awareness of rare diseases were generally low or inconsistent, regardless of country or professional group. In Spain, only around one-quarter of participants could correctly define rare diseases and orphan drugs, and most respondents prioritised funding for common diseases [59]. In China, only 5.3% of physicians reported moderate or good awareness, while 98.5% of emergency physicians rated their knowledge as minimal or insufficient [61]. Similarly, in Poland, 94.6% of physicians considered their knowledge insufficient or very poor, and fewer than 5% felt prepared to care for patients with rare diseases [72]. In Peru, over half of respondents demonstrated very poor knowledge, and fewer than half of physicians had heard the term “rare disease” [49]. Comparable findings were reported across Kazakhstan, Bahrain, Cameroon, and among dental professionals in Germany and Israel [55, 52, 51, 50, 65]. However, one study conducted in Mexico reported satisfactory levels of knowledge [69]. Despite this, the study also identified variability in awareness, perceptions, and attitudes between groups.

Variation was evident between professions and settings. Specialists and university-affiliated clinicians generally reported higher levels of awareness and knowledge than general practitioners or private-practice professionals. For example, Belgian specialists rated their education and awareness more positively than GPs, and university-based dentists in Germany achieved higher objective knowledge scores than those in other practice settings [16, 50]. Neurologists in Italy and in the global survey reported relatively high recognition of rare neurologic diseases as an important area of care, yet they still described substantial challenges in diagnosis, management, and access to resources [58, 62]. Likewise, Chinese physicians in a large national study showed better knowledge in relation to RD with which they were most familiar, but knowledge varied considerably across specific conditions [61].

The studies also showed that self-assessed competence did not always align with objective knowledge. Several papers highlighted discrepancies between what participants believed they knew and what they were able to demonstrate in questionnaires or quizzes. In Germany and Bahrain, for example, self-rated knowledge showed weak or inconsistent correspondence with actual performance [50, 52].

The role of RD education in undergraduate and postgraduate training was limited across most studies. Participants in multiple studies reported little or no formal teaching on RD during their professional preparation. In Spain, fewer than one-third of physicians had received RD training [59]. In Türkiye, only 7% of paediatric specialists and 3.8% of non-paediatric specialists considered time allocated to RD in medical education sufficient [63]. Nurses and nursing students in Poland similarly reported limited educational exposure but a strong desire for more teaching in this area [28]. Mexico and Brazil also highlighted the need for improved awareness and better integration of genetics-related knowledge into clinical education [66, 69].

Across all settings, the studies consistently identified multiple barriers contributing to poor knowledge and awareness of RD. These included the lack of formal education during undergraduate and postgraduate training, insufficient clinical exposure, and the absence of practice guidelines In China and India, most HCP relied on general search engines rather than specialist databases for information [53, 70], while in Belgium, few general practitioners (GPs) were aware of platforms like Orphanet [16]. Structural issues such as the lack of centralised national resources, e-learning tools, or patient registries also contributed to diagnostic delays and uncertainty in clinical decision-making. Self-assessed competence often did not align with objective knowledge scores, suggesting overconfidence or underestimation in certain professional groups [73].

Across studies, respondents consistently expressed a need for better educational resources, continuing professional development, and system-level support. Common recommendations included integrating RD content into medical, nursing, and dental curricula; developing case-based and interdisciplinary learning opportunities; improving access to specialist databases and information hubs; and producing clearer clinical guidelines and referral pathways. The multinational survey reinforced these findings, highlighting significant knowledge gaps, low diagnostic confidence, and the potential for educational interventions to reduce diagnostic delay and enable earlier referral and treatment [62]. Similarly, the study evaluating continuing medical education in China found that while baseline knowledge was very low, targeted education could improve awareness and some aspects of case analysis, even if overall gains remained modest [64].

Definitions of key constructs such as “knowledge,” “competence,” and “awareness” varied between studies, with few employing standardised or validated measurement instruments. There was a predominant reliance on self-reported data and there was considerable heterogeneity in questionnaire content, outcome measures, and analytic approaches which limits direct comparison of findings across contexts [74]. This heterogeneity makes direct comparison across countries and professions difficult, but the overall pattern remains clear: healthcare professionals and students across diverse settings often feel underprepared to recognise, diagnose, and manage RD, and they consistently report unmet educational and informational needs.

### Identifying themes

The studies were synthesised and then organised into key themes that reflect the review question and objectives of healthcare professionals’ knowledge and understanding of rare diseases. The work was completed using an inductive theme approach. This approach enhances transparency and rigor through structured coding, theme refinement and documentation, all of which facilitate clarity and reproducibility [75]. The themes identified included: Limited knowledge and awareness of rare disease,

### Limited knowledge and awareness of rare diseases

A prominent theme across the included studies was the overall low level of knowledge and awareness regarding rare diseases among healthcare professionals. This pattern was consistently observed across a wide range of professional groups, including medical students, nurses, general practitioners, dental practitioners, specialists, and allied health professionals, and across diverse geographical settings. Studies from multiple countries reported that many participants demonstrated insufficient factual knowledge of RD, including difficulties defining RD, understanding prevalence, and recognising specific conditions.

These knowledge deficits were consistent across the varying healthcare professionals. Studies involving nurses in Poland reported limited awareness and low preparedness to care for patients with RD, while dental professionals in Germany and Israel also demonstrated low or inconsistent knowledge, particularly in relation to recognising RD with orofacial manifestations [28, 50, 68].

Some variation was observed, Mexico reported relatively adequate knowledge levels among healthcare providers, although differences in awareness and perceptions between groups remained [69]. Similarly, a large national study in China found that physicians demonstrated better knowledge of RD with which they were more familiar, indicating that knowledge may be context-specific and influenced by prior exposure [61].

The Bahrain study highlighted a disconnect between self-perceived and actual knowledge, suggesting that healthcare professionals may overestimate their preparedness [69]. Nurses reported relying heavily on the internet for information, underlining the lack of structured and reliable educational resources in formal training [28]. Few studies examined the quality or reliability of these information sources, and it is possible that such reliance represents a pragmatic response to gaps in institutional support rather than a deficiency in professional practice.

These findings suggest that knowledge gaps are widespread across disciplines, healthcare systems and levels of clinical experience. Although variation in study design and measurement approaches may influence the extent to which these deficits are reported.

### The need for education and training among healthcare professionals

The second major theme identified across the studies was the insufficiency of formal education and training on rare diseases, which emerged as a critical and recurring concern. Participants frequently described feeling underprepared to identify, diagnose, and manage rare conditions, reflecting gaps in both undergraduate and postgraduate training.

Across multiple settings and professional groups, HCP reported limited exposure to RD within formal curricula. General practitioners, nurses and dental professionals consistently identified deficiencies in training, while specialists and university-affiliated clinicians reported relatively higher levels of exposure, suggesting variability based on training environment and experience.

There was a strong and consistent demand for improved education and training, including greater integration of RD into healthcare curricula, case-based learning approaches, and enhanced access to reliable information platforms. The multinational survey further emphasised the importance of targeted education in improving diagnostic confidence and reducing delays in care [62].

This study also identified an underuse of dedicated information platforms, suggesting a lack of awareness and integration of existing resources. In Kazakhstan and Peru, physicians acknowledged that medical education did not sufficiently prepare them for RD diagnosis and care and expressed strong support for including RD in mandatory assessments [63, 49]. Despite several studies noting underuse of information platforms, none explored the institutional or systemic barriers that might impede their integration into clinical training.

Overall, the evidence indicates that insufficient education and training represent a key and persistent barrier to effective RD recognition and management, highlighting the need for structured and accessible educational interventions.

### Inadequate preparedness and competence in managing rare diseases

Across the included studies, HCP consistently reported low levels of preparedness and competence in managing rare diseases, regardless of professional background or geographical setting.

Participants frequently reported difficulty recognising RD, understanding their aetiology and prevalence, and applying this knowledge in clinical practice. These challenges were observed across multiple regions, including Europe, Asia, the Middle East, and South America, where practicing clinicians described feeling insufficiently prepared to manage RD cases. In several studies, only a minority of participants reported confidence in their ability to provide appropriate care, and in some settings, awareness levels were particularly low, reinforcing the extent of this gap.

The tools used were often unvalidated and limited in scope, making it unclear whether they measured practical management competence or simply recall of epidemiological facts. Across all regions studied, participants expressed strong interest in receiving additional education, pointing to a critical unmet need in medical training and continuing professional development. This uniform expression in receiving education may be inflated by some social bias, particularly when the respondents are aware that the survey is linked to academic or healthcare institutions [76].

Several studies also highlighted a mismatch between perceived and actual competence, with weak or inconsistent correlations between self-assessed knowledge and objective performance. This suggests that HCP may not always accurately recognise their own limitations, which may have implications for clinical decision-making and patient safety [77].

The findings suggest that deficits in education and training translate directly into reduced clinical preparedness and confidence, highlighting a critical gap between theoretical knowledge and practical competence in RD care.

### Barriers contributing to poor knowledge and awareness

The studies consistently identified system level barriers contributing to poor knowledge and awareness of RD. These barriers extended beyond individual knowledge deficits and reflected broader structural limitations within healthcare systems.

These included a lack of formal education during undergraduate and postgraduate training, insufficient clinical exposure, and the absence of practice guidelines. In China and India, most HCP relied on general search engines rather than specialist databases for information [56, 53]. However, they did not assess the actual quality of information retrieved or whether this reliance resulted in suboptimal patient care, making it unclear whether the problem lies in the absence of guidelines or in the integration of existing resources into clinical practice.

A key issue was the limited availability and accessibility of specialised resources, including clinical guidelines, diagnostic tools, and dedicated information platforms. Structural issues such as the lack of centralised national resources, e-learning tools, or patient registries also contributed to diagnostic delays and uncertainty in clinical decision-making [16]. In addition, participants across multiple studies highlighted the lack of standardised clinical guidelines and decision-support tools, contributing to uncertainty in diagnosis and management. The absence of clear referral pathways and coordinated care systems further compounded these challenges, particularly for clinicians working in non-specialist or community settings.

These system-level challenges were closely linked to the diagnostic odyssey, with delayed or missed diagnoses often attributed to fragmented care pathways, limited specialist input, and inadequate access to reliable information [78]. As a result, HCP frequently reported uncertainty in clinical decision-making and reduced confidence in managing RD cases.

Across all regions, there was strong consensus on the need for improved system-level support, including better access to specialist resources, development of clinical guidelines, and integration of rare disease platforms into routine practice. However, few studies explored the underlying institutional or policy-level barriers that may hinder implementation of these solutions.

## Discussion

This scoping review highlights a consistent and global pattern of limited knowledge and awareness of rare diseases among HCP. However, beyond reiterating these findings, the review reveals important structural, methodological, and contextual issues that shape the current evidence base and have implications for practice, education, and policy.

### Differences across professional groups and healthcare systems

While variation in knowledge and awareness was observed across professional groups and healthcare settings, the ability to draw definitive comparisons is substantially constrained by the nature of the available evidence. Many included studies utilising heterogeneous samples, frequently combining healthcare professionals with students and, in some cases, members of the public. This aggregation may obscure meaningful differences in knowledge, roles, and clinical responsibilities [79].

Where more clearly defined samples were used, some patterns emerged. Specialists and university-affiliated clinicians demonstrated higher levels of knowledge compared to general practitioners, nurses, and community-based providers. These differences may reflect variation in exposure, access to specialist resources, and training environments, rather than true differences in baseline competence [80] There may be a greater engagement of specialist and academic clinicians with research activity, continuing professional development, and specialist networks, which can facilitate more frequent exposure to rare diseases and related knowledge resources [81]. However, the extent to which these factors account for observed differences remains unclear, as they are inconsistently measured across studies.

Across healthcare systems, similar limitations apply. Although studies from higher-income settings often reported underutilisation of specialist resources, and those from lower-resource settings highlighted structural barriers such as limited access to diagnostics and training, the heterogeneity of study designs and outcome measures makes direct cross-country comparison problematic. Furthermore, the absence of standardised assessment tools limits the ability to determine whether observed differences reflect genuine variation or methodological inconsistency [82]. While differences across professional groups and healthcare systems likely exist, they remain poorly characterised and insufficiently evidenced.

### Regional variability

The geographical distribution of studies across countries reflects an important but underexplored pattern. Much of the research originates from regions where rare disease policies, registries, or national strategies are emerging or established, such as Europe and parts of Asia [83, 84]. This may reflect greater institutional coordination, academic prioritisation, and policy-driven funding mechanisms that facilitate RD research, however, these factors were not directly examined in the included studies.

Additional factors may also contribute to this distribution. A possible explanation is that countries with more developed healthcare and research infrastructures may have established surveillance systems, patient registries, and academic networks, which enable the identification and recruitment of participants for RD studies [85, 86]. The presence of specialist centres and professional networks may increase clinician exposure to RD, thereby stimulating research activity. Publication and language bias may also play a role, as studies conducted in non-English-speaking or lower-resource settings may be underrepresented in indexed databases [87]. Finally, variations in medical education systems and research capacity may influence whether RD knowledge is formally assessed and studied.

Although differences in disease recognition and diagnostic capacity may create the impression of regional variation in prevalence, RD are globally distributed [88]. The absence of studies from countries with well-established healthcare systems, such as the United Kingdom and the United States, highlights an important gap in the evidence base. This lack of representation may reflect variation in research priorities, funding mechanisms, and publication patterns rather than differences in disease prevalence. In settings with limited diagnostic infrastructure, RD may remain underdiagnosed or misclassified, reducing their visibility within both clinical practice and research [89].

This concentration of evidence may introduce a significant geographical bias. As a result, the current evidence base may underestimate global inequities in RD knowledge, diagnostic capacity, and access to care.

### Broader implications of educational gaps

The findings extend to broader implications for clinical practice, patient outcomes, and health equity. Limited awareness among healthcare professionals contributes directly to delayed diagnosis, mismanagement, and fragmented care pathways [23]. These challenges reflect broader structural limitations within healthcare and training systems that remain predominantly focused on the more common diseases [18].

The review suggests that knowledge is not merely a cognitive issue but is closely linked to system-level factors, including the availability of clinical guidelines, access to specialist networks, integration of digital resources, and the structure of medical education. The persistent underuse of platforms such as Orphanet and specialist networks reflects a disconnect between available resources and clinical practice, rather than a complete absence of tools. Improving awareness and integration of platforms such as Orphanet and the European Reference Networks is essential to bridge the gap between available resources and clinical practice [1, 90].

The disparities in variation of knowledge across professional disciplines suggest that access to RD education and information is unevenly distributed. This reinforces the need for targeted interventions that account for professional context and level of experience. Furthermore, the association between increased knowledge and more supportive attitudes toward RD policy suggests that education may also influence broader healthcare priorities and advocacy.

### Methodological and sampling limitations of the evidence base

A key finding of this review is that the existing literature is both sparse and methodologically limited. Most studies employed cross-sectional survey designs using non-validated or partially validated instruments, with considerable heterogeneity in questionnaire content, outcome measures, and definitions of “knowledge” and “awareness”. This widespread reliance on ad hoc tools limits the reliability and comparability of findings across studies and contexts [91, 92].

The absence of standardised outcome measures prevents meaningful benchmarking between countries, professions, or educational interventions. Current research remains largely descriptive rather than evaluative, limiting the ability to determine what educational or system-level interventions are most effective.

The heavy reliance on self-reported data also raises concerns about measurement validity [76]. Several studies demonstrated discrepancies between perceived and actual knowledge, indicating that self-assessment alone is insufficient and may be influenced by cognitive biases such as overconfidence or underestimation [93].

The heterogeneity of included studies represents a critical limitation, particularly in relation to sampling strategies, and restricts the extent to which findings can be confidently attributed to specific professional groups. Engagement across professional groups was also uneven, with some studies heavily weighted toward physicians, while others included small or underpowered samples of nurses or allied health professionals. This imbalance limits the ability to draw profession-specific conclusions, despite clear evidence that knowledge levels, roles, and educational needs differ substantially across disciplines.

### Inequities in rare disease knowledge and education

An overarching finding of this review is the presence of systemic inequities in rare disease knowledge, education, and clinical preparedness, which appear to underpin many of the gaps and patterns identified across the evidence base. These findings align with wider literature highlighting the epistemic marginalisation of RD within healthcare education and research [94–96]. Despite their collective prevalence, RD are often deprioritised due to their individual rarity, resulting in their limited inclusion within curricula, research funding priorities, and clinical infrastructure.

Importantly, these inequities are not evenly distributed. They manifest across geographical regions, healthcare systems, and professional groups, reflecting disparities in access to education, specialist resources, and diagnostic capacity [25]. Healthcare professionals working in non-specialist or resource-constrained settings may experience compounded disadvantages, including limited training opportunities and reduced access to clinical guidance, which in turn can contribute to delayed diagnosis and fragmented care for patients.

The review also reinforces evidence that while educational interventions, such as targeted continuing professional development, can improve awareness, their impact may be limited if implemented in isolation. Without broader structural changes including integration into core curricula, improved access to specialist networks, and system-level prioritisation of RD, such interventions risk perpetuating existing inequities rather than resolving them. Addressing these disparities therefore represents not only an educational priority, but also a critical issue of health equity within RD care.

### Implications for research and practice

Several critical areas for future research have been identified. There is a need for the development and validation of standardised tools to assess RD knowledge and competencies across diverse healthcare professions and settings [97]. Longitudinal studies evaluating the impact of targeted educational interventions, such as curriculum integration, simulation training, or digital learning platforms, are also essential to identify best practices and determine long-term effectiveness.

Further qualitative research is warranted to explore the lived experiences of healthcare professionals managing RD cases, with a focus on diagnostic uncertainty, referral decision-making, and information-seeking behaviours [98, 99]. Additionally, implementation research should examine how national and regional policies, such as the UK Rare Diseases Framework or the European Reference Networks (ERNs), are influencing clinical practice and professional education on the ground [2, 100]. Finally, given the significant disparities observed between professional roles and across geographical contexts, research should prioritise inclusive, cross-disciplinary, and cross-national studies to develop scalable, equitable strategies for RD training and knowledge dissemination.

### Implications for policy

There are important implications for RD policy across the globe. In the UK, the results support urgent implementation of commitments outlined in the UK Rare Diseases Framework [101], which emphasises improving healthcare professional awareness, early diagnosis, and patient care coordination. Despite this strategic direction, the persistent lack of knowledge among frontline clinicians highlights a need for actions, such as embedding RD into General Medical Council-approved medical curricula, postgraduate training pathways, and professional revalidation processes.

Across Europe, the European Reference Networks (ERNs) play a central role in coordinating cross-border care and knowledge sharing. European health policy must focus not only on building centralised resources, but also on increasing accessibility and practitioner engagement through language localisation, professional incentives, and integration into electronic health systems [3]. Additionally, the EU Pharmaceutical Strategy, must be paired with investments in clinician education on RD, to ensure that innovation translates into clinical impact [102].

Globally, the review reinforces the need for WHO member states to recognise rare diseases as a global public health challenge, particularly in low- and middle-income countries where structural barriers to training and information access are greatest. Policies should support the creation of national RD registries, foster international data sharing, and build localised digital platforms. This requires coordinated policy action that integrates education, digital infrastructure, and clinical support systems, rather than relying on isolated educational initiatives.

### Strength and limitations of the review

This review synthesises evidence across multiple regions and professional groups, providing a comprehensive and interdisciplinary overview of RD knowledge and education. By incorporating studies from diverse healthcare systems, it captures a wide range of geographical and economic contexts, enhancing the generalisability and policy relevance of the findings [35].

Methodologically, the review was conducted in accordance with the PRISMA-ScR framework, using a comprehensive multi-database search strategy and a transparent study selection process, which strengthens its rigour and reproducibility.

However, as is characteristic of scoping review methodology, no formal quality appraisal of included studies was undertaken, with the focus placed on breadth rather than critical evaluation [33]. As a result, the findings should be interpreted with caution, as the methodological limitations of the included studies may influence the overall strength and reliability of the evidence.

## Conclusion

This scoping review demonstrates that healthcare professionals globally remain insufficiently prepared to recognise, diagnose, and manage rare diseases, reflecting persistent gaps in knowledge, education, and access to specialist resources. These deficits are not uniformly distributed, but instead reflect broader structural inequities across healthcare systems, professional roles, and geographical contexts. Addressing these challenges requires coordinated, system-level action that integrates education, clinical support, and policy development.

Based on the findings of this review, four key recommendations are proposed. First, rare disease education should be embedded as a mandatory component of undergraduate medical, nursing, and allied health curricula, ensuring that all healthcare professionals develop baseline competencies in recognising and responding to rare conditions. Second, there is a need to expand accredited continuing professional development programmes, including flexible, accessible online learning platforms, to support ongoing knowledge development across the workforce. Third, healthcare systems should prioritise the integration and promotion of existing digital resources, including Orphanet and the European Reference Networks, to facilitate timely access to reliable information and specialist support in clinical practice. Fourth, national and international policy frameworks should support the development of coordinated rare disease strategies, including investment in registries, clinical guidelines, and referral pathways to reduce diagnostic delays and improve care coordination.

These actions are essential to strengthen the capacity of healthcare systems to respond to rare diseases and to ensure more equitable access to knowledge, diagnosis, and care for affected individuals and families.

## Supplementary Information

Below is the link to the electronic supplementary material.

Supplementary Material 1

## Data Availability

All data generated or analysed during this study are included in this published article [and its supplementary information files].
