# Supplementary material for: Knowledge and awareness of rare diseases among healthcare professionals: a scoping review
Source: Orphanet J Rare Dis. 2026 Aug 13;21:409. doi: 10.1186/s13023-026-04520-1 (PMC13757078; doi:10.1186/s13023-026-04520-1)
Supplement: Supplementary file 1 [file 13023_2026_4520_MOESM1_ESM.docx]

Additional Files

**Additional File 1. Search Strategy**

- Developed using the Population–Concept–Context (PCC) framework
- Population: Healthcare professionals (e.g., physicians, nurses, dentists, allied health)
- Concept: Knowledge, awareness, understanding, education, training
- Context: Rare diseases (including orphan diseases and rare conditions)

**Databases Searched**

- PubMed/MEDLINE
- CINAHL (EBSCOhost)
- Scopus

**Search Dates**

- Databases searched from inception to March 2026
- Final search conducted in March 2026

**Search Process (JBI Three-Step Strategy)**

1.Initial limited search conducted in PubMed to identify keywords and index terms

2.Comprehensive search performed across all databases using identified terms

3.Reference lists of included studies screened for additional relevant articles

**Search Terms and Boolean Operators**

- Search terms were grouped into three key concepts:
  - Healthcare professionals
  - Knowledge and awareness
  - Rare diseases
- Terms within each concept were combined using **OR**
- Concepts were combined using **AND**

**PubMed / MEDLINE Search String**

("healthcare professional*" OR "health personnel" OR clinician* OR physician* OR doctor* OR nurse* OR dentist* OR "allied health")
AND
(knowledge OR awareness OR understanding OR education OR training OR competence)
AND
("rare disease*" OR "orphan disease*" OR "rare condition*")

**CINAHL (EBSCOhost) Search String**

(MH "Health Personnel" OR "healthcare professional*" OR clinician* OR physician* OR nurse* OR dentist* OR "allied health")
AND
(knowledge OR awareness OR understanding OR education OR training OR competence)
AND
("rare disease*" OR "orphan disease*" OR "rare condition*")

**Scopus Search String**

TITLE-ABS-KEY ("healthcare professional*" OR "health personnel" OR clinician* OR physician* OR nurse* OR dentist* OR "allied health")
AND
TITLE-ABS-KEY (knowledge OR awareness OR understanding OR education OR training OR competence)
AND
TITLE-ABS-KEY ("rare disease*" OR "orphan disease*" OR "rare condition*")

**Grey Literature Search**

- Google Scholar searched using combinations of: “rare diseases” AND “healthcare professionals” AND “knowledge”
- Organisational websites searched: Orphanet, EURORDIS and Department of Helath and Social Care.

**Study Selection Support**

Forward citation searching was conducted using Google Scholar for all included studies. Titles and abstracts of citing articles were screened against the inclusion criteria.

**Additional File 2. PCC Framework for search terms.**

| **PCC Framework** | **Search Terms** |
| --- | --- |
| **Population –** Healthcare Professionals | Healthcare Professional  Health personnel  Healthcare Worker  Clinician  Nurse |
| **Concept –** Knowledge and Awareness | Knowledge  Awareness  Understanding  Education |
| **Context** – Rare Diseases | Rare Disease  Orphan Disease  Rare Condition |

**Additional File 3. Inclusion/exclusion criteria**

| **Criteria Type** | **Inclusion Criteria** | **Exclusion Criteria** |
| --- | --- | --- |
| Population | Healthcare professionals (including doctors, nurses, dentists, allied health staff) | Patients, carers, students only, general public |
| Concept | Knowledge, awareness, understanding, education related to rare diseases | Focus on treatment, diagnosis, or management of rare diseases  Focus on specific rare diseases |
| Context | Clinical settings, healthcare training, continuing professional development | Non-healthcare settings (including schools, public campaigns) |
| Study Design | qualitative, quantitative, mixed methods, reviews | Editorials, opinion pieces, commentaries, letters |
| Publication Type | Peer-reviewed articles, grey literature, theses/dissertations | Conference abstracts without full papers, blog posts, promotional materials |
| Availability | Full-text available | Full-text not accessible |

**Additional File 4. PRISMA-ScR flow diagram**

**Additional File 5 – Data Extraction Table**

| Study | Author | Year | Country | Aims | Population | Methods | Outcome | Key Findings |
| --- | --- | --- | --- | --- | --- | --- | --- | --- |
| Information needs of physicians regarding the diagnosis of rare diseases: a questionnaire-based study in Belgium | Vandeborne et al | 2019 | Belgium | To investigate the information needs of physicians concerning the diagnosis of rare diseases (RDs) and to assess their awareness and utilisation of RD-specific information resources | 295 Physicians practicing in Belgium, including general practitioners (GPs), paediatricians, and other specialists | Quantitative design: Cross Sectional Survey. | Level of knowledge and awareness of RDs among physicians.  Self-assessed adequacy of academic and ongoing medical education on RDs.  Frequency and sources of information used when encountering potential RD  Awareness/ utilisation of RD platforms | GPs exhibited the lowest levels of RD knowledge and awareness, while specialists demonstrated higher levels.  GPs rated their academic education on RDs as insufficient, whereas paediatricians and other specialists rated it significantly better. |
| Needs assessment study of rare diseases education for nurses and nursing students in Poland | Walkowiak and Domaradzki | 2020 | Poland | To assess the educational needs and current level of awareness about rare diseases among Polish nurses and nursing students. | 142 nurses  113 nursing students | Quantitative design: Cross Sectional Survey. | knowledge of rare diseases, preparedness to care for patients with rare diseases, and attitudes towards rare disease education. | Majority of nurses reported limited knowledge about rare diseases.  Many respondents felt unprepared to care for patients with rare conditions.  There was high interest in receiving further education and training on this topic. |
| Study to Investigate the Knowledge of Rare Diseases among Dentists, Orthodontists, Periodontists, Oral Surgeons and Craniomaxillofacial Surgeons | Kühne et al | 2021 | Germany | To assess the level of knowledge about rare diseases among dentists and compare self-assessed versus actual knowledge | 267 dentists: 64.0% general, 25.5% specialist, 10.5% university-based | Quantitative design: Cross Sectional Survey. | Objective knowledge test scores and self-assessed knowledge ratings. | University-based dentists had significantly higher objective knowledge about rare diseases.  All groups had low self-assessed knowledge, indicating a general underestimation of knowledge gaps |
| Rare disease awareness and perspectives of physicians in China: a questionnaire-based study | Li et al | 2021 | China | To assess the awareness and perspectives of physicians in China regarding rare diseases (RDs), identify factors influencing their awareness, and gather expert opinions on improving RD awareness | 224 physicians from various hospitals across China  9 rare disease experts interviewed using open-ended questions. | Multi-method study: questionnaire and expert interviews.  Cross sectional survey to physicians RDs.  Interviews; with 9 RD experts. | Only 5.3% of physicians were moderately or well aware of RD  80.1% had suspected RD in patients fewer than three times.  Experts emphasised the necessity of RD awareness for early diagnosis and timely treatment.  Recommendations included enhancing medical school education and establishing an online information hub for RD | Only 5.3% of physicians were moderately or well aware of RD  80.1% had suspected RD in patients fewer than three times.  Experts emphasised the necessity of RD awareness for early diagnosis and timely treatment.  Recommendations included enhancing medical school education and establishing an online information hub for RD |
| Are rare diseases overlooked by medical education? Awareness of rare diseases among physicians in Poland: an explanatory study | Walkowiak and Domaradzki | 2021 | Poland | To assess the awareness and knowledge of rare diseases (RDs) among Polish physicians, evaluate their self-assessed competence in managing RDs, and gather their opinions on the inclusion of RDs in medical education curricula. | 165 medical doctors undergoing specialization courses at the Poznan University of Medical Sciences, Poland | Quantitative design: Cross Sectional Survey. | Level of knowledge about RDs among physicians.  Self-assessed preparedness to care for RD patients.  Opinions on the necessity of mandatory RD courses in medical education.  Sources of information utilized for RD knowledge. | Most physicians lacked basic knowledge about the etiology, epidemiology, and prevalence of RDs.  Many had difficulty distinguishing RDs from more common disorders.  94.6% perceived their knowledge on RDs as insufficient or very poor.  Less than 5% felt prepared to care for patients with RDs.  Over 83% believed that RDs constitute a serious public health issue.  17% opined that mandatory courses on RDs are not necessary in curricula |
| Knowledge level of medical students and physicians about rare diseases in Lima, Peru | Flores et al | 2022 | Peru | To assess the knowledge level of medical students and physicians about rare diseases | 720 participants  338 medical students  382 physicians | Quantitative design: Cross Sectional Survey. | Awareness and knowledge levels in rare diseases.  Attitudes about training and curricular inclusion.  Perception of rare diseases as public health issue. | 48.7% of physicians had heard of the term rare disease.  Over half the participants demonstrated very poor knowledge of rare diseases.  Most respondents had not received training on rare diseases. |
| Awareness and Knowledge of Rare Diseases in German Dentists, Dental Specialists and Oral and Maxillofacial Surgeons: A Country-Wide Survey | Benz et al | 2022 | Germany | To evaluate the knowledge and management of rare diseases and their orofacial alterations by dentists, dental specialists and oral and maxillofacial surgeons and dentists | 309 dentists and oral and maxillofacial surgeons in private practice and 18 dentists or oral and maxillofacial surgeons working at universities | Quantitative design: Cross Sectional Survey. | Perceived clinical relevance of rare diseases.  Formal exposure, such as education or lectures on RD.  Active research involvement in RD.  Self-rated knowledge levels, | Private Practice Clinicians - Most considered RD relevant for clinical practice  University-based Practitioners - No training in 77% of participants  Self-Assessment - The majority rated their knowledge as inadequate, with some claiming it to be “very good” |
| Poor education and urgent information need for emergency physicians about rare diseases in China | Zhou et al | 2022 | China | To investigate Chinese emergency physicians' basic knowledge, information access, and educational needs regarding rare diseases (RDs). | 539 emergency physicians across China. | Quantitative design: Cross Sectional Survey. | Accuracy in estimating RD prevalence.  Self-assessed knowledge levels about RDs.  Preferred sources for obtaining RD information.  Perceived barriers to RD diagnosis. | 4.27% of respondents correctly estimated the prevalence of RDs.  A minimal number were aware of the exact number of RDs listed in China's first official list in 2018.  98.5% rated their knowledge about RDs as minimal or insufficient. |
| The Awareness of Rare Diseases Among Medical Students and Practicing Physicians in the Republic of Kazakhstan. An Exploratory Study | Walkowiak et al | 2022 | Kazakhstan | To assess the awareness and knowledge of rare diseases (RDs) among medical students and practicing physicians, evaluating their preparedness to care for RD patients and identifying gaps in medical education related to RDs. | 308 individuals comprising:  207 medical students  101 practicing physicians | Quantitative design: Cross Sectional Survey. | Level of knowledge about RDs  Self-assessed preparedness to care for RD patients  Awareness of RD-related healthcare policies  Perception of RDs as a public health issue  Sources of information on RDs utilised | Only 9.9% of physicians and 12.1% of students rated their knowledge about RDs as sufficient.  The majority lacked knowledge about the etiology, epidemiology, and prevalence of RDs.  Many had difficulty distinguishing RDs from more common disorders. |
| A survey of awareness of diagnosis and treatment of rare diseases among healthcare professionals and researchers in India | Vashishta et al | 2023 | India | To assess the awareness and understanding of the diagnosis and treatment of rare diseases among healthcare professionals and researchers | 52 Healthcare professionals and 65 researchers across India. | Quantitative design: Cross-sectional survey. | Level of awareness about rare diseases.  Understanding of diagnostic procedures for rare diseases.  Knowledge of treatment options. | The study found varying levels of awareness and understanding among healthcare professionals and researchers regarding rare diseases. |
| Knowledge and Awareness of Rare Diseases Among Healthcare Professionals in the Kingdom of Bahrain | Sinan et al | 2023 | Kingdom of Bahrain | To assess healthcare professionals' general awareness and knowledge of rare diseases (RDs) in a tertiary hospital in the Kingdom of Bahrain | 333 HCPs comprising:  25.2% physicians, 53.8% nurses, 21.0% allied health personnel | Quantitative design: Cross-sectional survey. | Awareness of the term "rare diseases."  Ability to recognise age groups frequently affected  Knowledge of common causes of RDs worldwide.  Correlation between self-declared knowledge and actual knowledge | 87.4% of participants had heard the term "rare diseases" prior to the survey.  Participants could identify age groups frequently affected by RDs and common causes of RDs worldwide  Only four participants answered all knowledge questions correctly.  Weak correlation between self-declared knowledge and overall quiz. |
| Exploring Healthcare Practitioners’ Knowledge of Rare Diseases in Cameroon | Ngoumou et al | 2023 | Cameroon | To examine the knowledge of Cameroonian physicians on rare diseases (RDs) to highlight the medical constructions of RDs in Cameroon. | 23 physicians from various medical institutions across the country. | Quantitative design: Cross-sectional survey (open and closed items). | Level of theoretical knowledge about RDs  Frequency of encountering RD cases in clinical practice.  Identification of common RDs observed.  Challenges faced in diagnosing and managing RDs | 40.9% of participants correctly identified that a rare disease has a very low prevalence.  73.1% had encountered at least one case of a rare disease in their practice.  81% reported difficulties in detecting rare diseases, due to limited resources and lack of training. |
| Awareness and Care Practices for Rare Neurologic Diseases among Senior Neurologists: A Global Survey | Janga et al | 2025 | Global - Africa, the Americas, Asia/Oceania, and Europe, | Evaluated awareness and current care status of RNDs among esteemed neurologists affiliated with the World Federation of Neurology (WFN). | 190 neurologists of which 64 responded (34% response rate). | Quantitative design: Cross sectional Survey (34 items) | To evaluate awareness and current diagnosis and care practices for RNDs. | 89% agreed that patients with rare neurological diseases should receive timely and effective care equivalent to that provided for more common conditions.  77% believed that most RNDs could be accurately diagnosed in their country.  However, cross‑country variation in RND diagnosis and care, shaped in part by socioeconomic context, points to inequities in clinical resources and preparedness exist. |
| Enhancing Rare Disease Awareness and Education Among Medical Professionals and Students in Türkiye | Karagöz et al | 2025 | Türkiye | Assessment of the knowledge, awareness, and  educational needs related to RDs among 5th‐ and 6th‐year medical students at Atatürk University | 398 responses submitted (79.6% response rate), comprising of  258 physicians and  132 medical students | Quantitative design: Cross Sectional Survey (comprised of 6 sections) | Examination of demographics, self‐assessed knowledge, awareness, and perceptions of RD‐related education. | Paediatric specialists demonstrated significantly higher self‑assessed knowledge of rare diseases compared with non‑paediatric specialists. Nonetheless, substantial gaps persisted in both groups, especially in awareness of RD prevalence and diagnostic timelines exist. |
| Effects of continuing medical education on emergency trainees’ rare disease knowledge and attitude: a single-center study | Liu et al | 2025 | China | Evaluate the effects of continuing medical education (CME) on the knowledge and attitude of emergency physicians | 228 emergency physicians of which 218 returns (95.6% response rate), of which 108 received RD CME training and 110 did not receive RD CME training. | Quantitative design: Cross Sectional Survey (30 items). | Demographic data, knowledge, and attitudes regarding RDs. | 98.2% reported insufficient RD knowledge.  CME participation was associated with greater awareness of RD and better case‑analysis ability, though it produced only minor gains in familiarity with RD professional websites. |
| Nurses’ Knowledge of Rare Diseases: A Systematic Review | Munoz Sanchez et al | 2025 | n/a | To update the existing  knowledge on nurses’ level of understanding regarding rare diseases. | From 191 papers identified, 24 studies were included | Evidence Synthesis: Systematic review of Scopus, PubMed, CINAHL, SciELO, and Cochrane  Library, selecting studies published between 2014 and 2024. | Rare disease knowledge among Nurses | Across the 24 included studies, there was a consistent and substantial gap in nursing education on rare diseases, indicating that current curricula and training pathways do not adequately prepare nurses to recognise, manage, or support individuals with rare conditions. |
| Education and information needs for physicians about rare diseases in Spain | Ramalle-Gómara et al | 2020 | Spain | To report the training needs and the  perceived shortcomings of Spanish physicians of the public health system in the diagnosis, treatment and monitoring of patients with rare diseases | 264 physicians of which 169 returns (64,0% response rate  (132 (78.1%) from primary care and 37 (21.9%) from a hospital setting. | Quantitative design: Cross Sectional Survey (26 items). | Training needs  in the diagnosis, treatment and  monitoring of patients with rare diseases | Fewer than one‑third of physicians had received rare‑disease training during their undergraduate or postgraduate education, with hospital‑based professionals more likely to receive such training at the postgraduate stage. |
| Educational needs in diagnosing rare diseases: A multinational, multispecialty clinician survey | Rohani-Montez et al | 2023 | US and European Union Medscape member clinicians. | To assess clinician knowledge, experience, and educational  needs in RDs | 69,317 clinicians of which 978 returns  who specialize in 16 different therapeutic areas completed  the survey (1.4% response rate); comprising  927 (94.8%)  physicians  51 (5.2%) nurses or advanced  practice nurses. | Quantitative design: Cross Sectional Survey (14 items). | Knowledge, experience, and educational  needs | Rare diseases were widely underestimated. Two‑thirds believing they are 50–500 times rarer than standard definitions.  59% reported never or rarely encountering RD patients.  87% had participated in an RD diagnosis, only 19% felt mostly or very confident doing so.  Diagnostic barriers were frequently reported (38–44%), including limited knowledge of RD signs and symptoms, insufficient time for investigation, lack of guidelines, restricted test access, and referral challenges.  Short online RD education was recommended. |
| Knowledge and attitudes about rare genetic diseases among practitioners of oral medicine pathology in Brazil: a cross-sectional study | Trezena et al | 2025 | Brazil | To analyse the knowledge and attitudes of  Brazilian Oral Medicine and Pathology (OM/OP) specialists about genetic  diseases | 273 specialists, members of the Brazilian Society of  Stomatology and Oral Pathology (SOBEP), 58 OM/OP specialists return (21.2% response rate) | Quantitative design: Cross Sectional Survey (14 items). | Knowledge and attitudes | 67.2% reported attending theoretical courses on diagnosing and genetic testing for genetic diseases  79.3% identified barriers to integration between Medical Genetics and OM/OP.  Longer PhD‑level experience was significantly associated with better knowledge.  The most common RD recalled was dental abnormalities, tumour‑related findings, and Gorlin–Goltz, Gardner syndrome, and neurofibromatosis |
| Rare diseases and orphan medicines: knowledge and perceptions of healthcare professionals and the public | Vassallo et al | 2022 | Malta | to assess the public’s and healthcare professionals’ awareness and perception on rare diseases and orphan  drugs, and to identify challenges faced by both populations | 228 members of the public (27 were rare disease pateints) and 73 healthcare professionals. No response rate reported. | Quantitative design: Two Cross Sectional Surveys one for healthcare professionals and one for the public. | experience and awareness of the public and healthcare professionals about rare diseases and orphan medicines | Among the public  -37% knew or were related to someone with a rare disease,  -12% were themselves rare‑disease patients.  -19% of patients had experienced difficulties obtaining an orphan drug.  Among healthcare professionals  -55% had diagnosed, encountered, or examined a rare‑disease patient during their career  -32% had dispensed or attempted to dispense an orphan drug.  -27% reported encountering problems when trying to obtain an orphan drug for patients. |
| Physicians’ knowledge on specific rare diseases and its associated factors: a national cross‑sectional study from China | Zhang et al | 2022 | China | To assess Chinese physicians’ knowledge on specific rare diseases and identify its associated factors | 4387 physicians | Quantitative design: National Cross Sectional Survey (102 items). | Knowledge of 34 specific rare diseases and identify its associated factors | 66.16% achieved a total knowledge score of 2 or 3.  For 10 rare diseases (29.4%), the median knowledge score was ≤1.5, indicating substantial gaps in disease‑specific understanding.  Higher knowledge scores were independently associated with female physicians (β = 0.08, p < 0.05) and those with *monthly incomes of 5,000–10,000 RMB (β = 0.11, p < 0.01) or *10,000–30,000 RMB (β = 0.14, p < 0.05).  Physicians in internal medicine, obstetrics and gynaecology, radiology, intensive care, and surgery also demonstrated relatively higher scores. |
| Knowledge and Associated Factors about Rare Diseases among Dentists in Israel: A Cross-Sectional Survey | Mijiritsky et al | 2021 | Isreal | to assess levels of knowledge among Israeli dentists about  rare diseases with orofacial manifestations, and whether occupational, regional and social factors  influence those levels | 309 Dentists | Quantitative design: Cross Sectional Survey | Knowledge and associated factors | Respondents correctly identified the prevalence of RD.  Many underestimated the proportion of rare diseases with orofacial manifestations  70.1–95.2% had previously treated a patient with a RD.  Findings indicate clinical exposure and diagnostic knowledge may not align.  Knowledge levels were reported to be influenced by a range of socio-demographic factors. |
| Awareness of rare and genetic neurological diseases among Italian neurologist: A national survey | Mancuso et al | 2020 | Italy | to quantitatively describe the awareness of RNDs among the neurological  community of the Italian Society of Neurology (SIN) | 2500 neurologists, of which 104 neurologists’ participants (4% response rate) | Quantitative design: Cross Sectional Survey. | Describe  awareness of RNDs | 92% had a special interest in RNDs.  53% reported that most of the RNDs may be  easily diagnosed.  80% were aware of the coordinated care across their Regions  67% consider collaboration  with pharma industries as an important.  75% declared that was possible to perform a complete work up for RNDs; however,  this percentage varies among the different RNDs  62% prescribe orphan  drugs, and in 75.7% of cases the clinical follow-up of RNDs  patients occur every 6 months. |
| General knowledge and opinion of future health care and non-health care professionals on rare diseases | Ramalle-Gómara et al | 2015 | Spain | To evaluate the general knowledge and opinion on RD of future health care and non-health care professionals. | 234 responses. The sample comprised of  -48 resident doctors (RDr; 28 general practitioners and 12 RDr belonging to other specialities)  - 102 2^nd^ and 3^rd^ year nursing students  -45 students of technical and vocational health education  -40 non-health-related university students | Quantitative design: Cross Sectional Survey. | General knowledge on RD; prioritization of research of RD; willingness to assign resources to RD and real distribution of resources to RD | 25% correctly identified the definitions of RD and orphan drugs.  Attitudes toward resource allocation showed no significant differences between study groups.  73% believed healthcare funding should prioritise common diseases.  Resident doctors lacked experience and time to diagnose and manage RDs. |
| Assessing knowledge, perceptions, awareness and attitudes on rare diseases among health care providers and health students in Mexico | Gómez-Díaza et al | 2023 | Mexico | To assess the degree of knowledge, perceptions and attitudes of healthcare practitioners and students. | 944 responses from health care providers (including medicine) and students (undergraduate, graduate and postgraduate) | Quantitative design: Cross Sectional Survey. | Knowledge, perceptions, awareness and attitudes and it was aimed to measure basic knowledge | Knowledge levels differed significantly by academic degree, with the postgraduate group scoring highest (Kruskal–Wallis: KW = 50.5, d.f. = 2, p < 0.001).  Scores for perceptions, awareness, and attitudes varied significantly across careers in both undergraduate and graduate groups, with Medicine consistently achieving the highest scores (p < 0.001 for undergraduates; p = 0.03 for graduates). |
